# Hepatitis C virus p7 regulates Core protein processing via signal peptide peptidase to promote virion assembly

**DOI:** 10.1128/jvi.00288-26

**Published:** 2026-04-17

**Authors:** Ming-Jhan Wu, Patrick Healy, Chia-Wen Chang, Christoph Welsch, MinKyung Yi

**Affiliations:** 1 Department of Microbiology and Immunology, University of Texas Medical Branch at Galveston 12338, Galveston, Texas, USA; 2 Department of Internal Medicine I, Goethe University Frankfurt, University Hospital, Frankfurt/Main, Germany; 3 Molecular Hepatology & Inflammation Research, Goethe University Frankfurt, University Hospital, Frankfurt/Main, Germany; Wake Forest University School of Medicine, Winston-Salem, North Carolina, USA

**Keywords:** signal peptide peptidase (SPP), hepatitis C virus, Core, p7

## Abstract

The hepatitis C virus (HCV) p7 protein is a viroporin essential for the production of infectious virus particles and has been implicated in regulating Core protein localization. Here, we identify a previously unrecognized role of p7 in modulating Core maturation through interaction with host signal peptide peptidase (SPP), a mechanism that remains intact even in ion-channel deficient mutants. Using a transient expression system, we show that p7 from genotype 1a (H77S) delays SPP-mediated cleavage of full-length Core (fl-Core) to mature Core (m-Core), while genotype 2a (JFH1) p7 exerts a weaker inhibitory effect. Both p7 variants bind endogenous SPP, but JFH1 p7 shows reduced affinity, correlating with diminished inhibition of Core processing. A conserved leucine-rich motif (L51–55) in the p7 C-terminus is essential for SPP binding; its mutation disrupts Core processing and markedly reduces infectious virus production. Moreover, fl-Core preferentially associates with E1 glycoprotein compared to m-Core, supporting its role in virion assembly. These findings uncover a mechanism by which p7 regulates the timing of Core maturation via SPP interaction to promote efficient HCV particle assembly.

**IMPORTANCE:**

Hepatitis C virus (HCV) assembly depends on the tightly regulated coordination of viral structural proteins and host factors. While p7 is well-known for its viroporin activity, its role in viral protein maturation has remained unclear. This study uncovers a novel function of p7 in modulating Core protein processing via interaction with host signal peptide peptidase (SPP). We identify a conserved leucine-rich motif in p7 essential for this interaction and demonstrate that delayed Core maturation enhances Core-E1 association and promotes efficient virus assembly. These findings expand our understanding of HCV morphogenesis and highlight the p7-SPP axis as a potential target for antiviral intervention.

## INTRODUCTION

Hepatitis C virus (HCV) is a significant global health concern, affecting approximately 70 million individuals worldwide, potentially leading to liver cirrhosis and hepatocellular carcinoma (1). It is a positive-sense single-stranded RNA virus of the Flaviviridae family (2), encoding a polyprotein cleaved into structural (Core, E1, and E2) and non-structural (p7, NS2, NS3, NS4A, NS4B, NS5A, and NS5B) proteins (3, 4). Among these, p7, a small integral membrane protein, plays a crucial role in viral assembly and release by modulating intracellular pH and ion homeostasis (5).

The p7 protein, a viroporin, approximately 63 amino acids (aa) in length, functions as a virus-encoded ion channel. It is comprised of two transmembrane domains connected by a short cytoplasmic loop, which enables its insertion into intracellular membranes such as the endoplasmic reticulum (ER) (6). Functionally, p7 plays a critical role in HCV particle assembly, maturation, and secretion. It has been shown to protect nascent virions from acidification within the secretory pathway, thereby facilitating the production of infectious virus particles (5).

**Fig 1:**
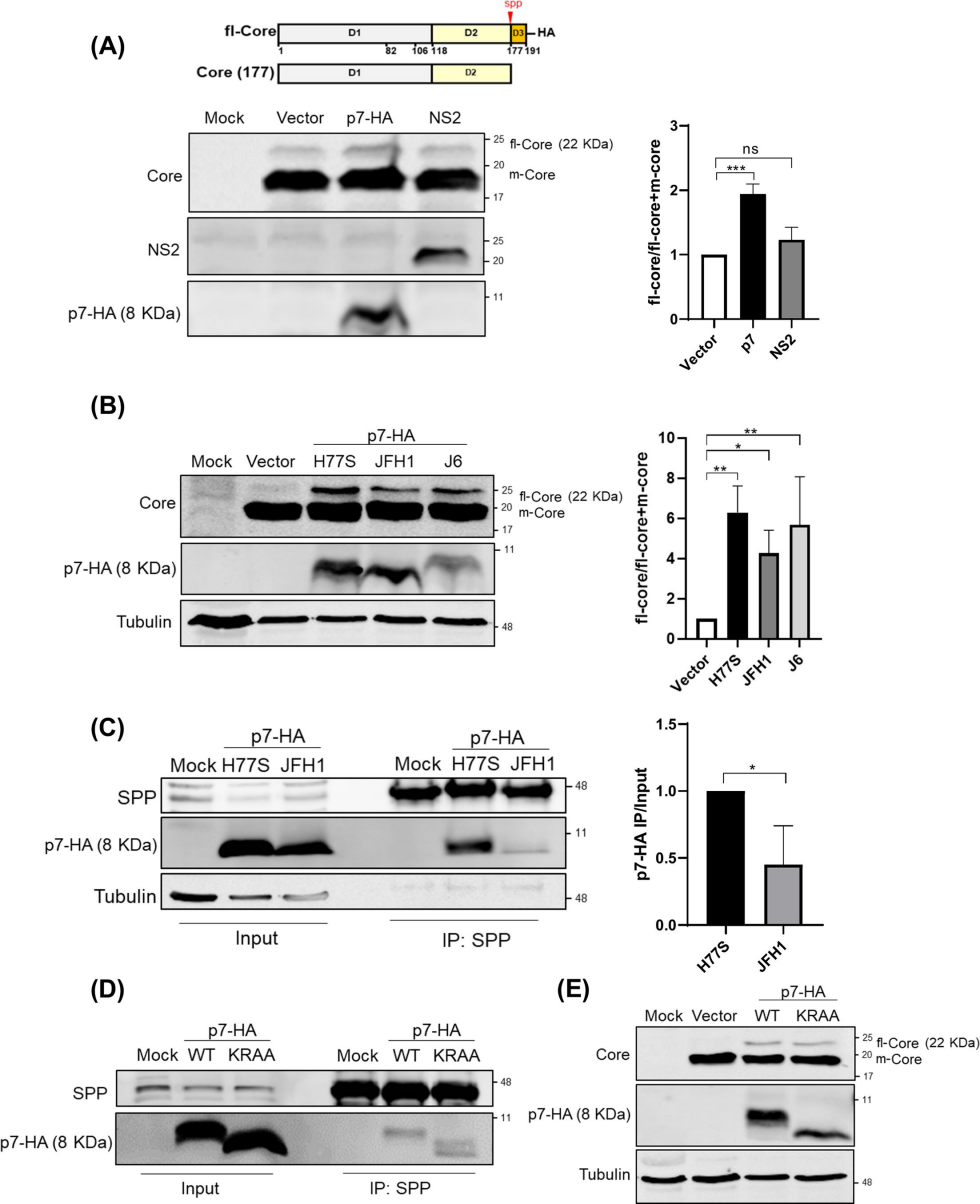
HCV p7 delays Core protein processing through interaction with SPP. (**A**) Schematic of full-length Core (fl-Core) tagged with HA at the C-terminus. The SPP cleavage site (red) is located just upstream of Domain 3 (D3), resulting in the mature Core (1–177 aa) (m-Core), which lacks D3. Western blot analysis shows fl-Core and Core-177 levels following co-expression with empty vector, p7-HA, or NS2 from H77S. Quantification of fl-Core as a percentage of total Core is shown (right; mean ± SD, *n* = 3). (**B**) Western blot showing Core processing with HA-tagged p7 from H77S, JFH1, and J6. fl-Core levels are quantified (right; mean ± SD, *n* = 3). (**C**) Co-immunoprecipitation (co-IP) of HA-tagged p7 (H77S or JFH1) with endogenous SPP in FT3-7 cells. (**D**) Co-IP of HA-tagged wild-type p7 or p7-KRAA mutant with endogenous SPP. SPP was detected by Western blot. (**E**) Western blot showing Core processing with HA-tagged wild-type p7 or p7-KRAA mutant. Statistical significance was determined by unpaired *t*-test: ****, *P* < 0.0001; ***, *P* < 0.001; **, *P* < 0.01; ns = not significant *P* > 0.05.

The core protein is a highly conserved structural protein that forms the viral nucleocapsid and plays multiple roles in the virus life cycle across different HCV genotypes. The full-length Core (fl-Core) encodes three domains (D1, D2, and D3) (Fig. 1A) (7), processed by the signal peptide peptidase (SPP) in the ER membrane to a mature Core (m-Core), encoding D1 and D2, which then associates with the lipid droplets (LDs) in a D2-dependent manner (8–10). These Core-LD associations serve as assembly platforms for HCV particles. Mutations disrupting this interaction lead to rapid proteasomal degradation of Core, underlining the structural and functional importance of D2 (8, 11). Domain 3 (D3), located at the Core C-terminus, functions as a signal sequence that anchors the full-length Core to intracellular membranes: ER and mitochondria-associated membranes (12). Following translation, the Core protein is initially embedded in the ER membrane via D3. SPP is an intramembrane-cleaving aspartyl protease with nine transmembrane domains. It recognizes Core and cleaves between residues 176 and 177, thereby releasing m-Core into the cytoplasm (11).

SPP belongs to the GxGD-type family of intramembrane-cleaving proteases, which also includes the presenilins responsible for γ-secretase activity. These proteases mediate regulated intramembrane proteolysis (RIP), a conserved cellular mechanism that sequentially processes transmembrane proteins to modulate their function or release signaling fragments (13). In the context of HCV infection, SPP-mediated cleavage of Core represents a critical step in regulating the distribution of Core between membrane-associated and cytoplasmic pools, a balance that directly impacts viral assembly and infectivity. LDs serve as platforms for virus assembly, and HCV strain-dependent in Core localization to LDs can influence the efficiency of this process. Notably, p7 has been shown to redirect Core to the ER even when ion channel-deficient mutants of p7 are used, although the underlying mechanism remains unclear (14).

In this study, we investigated the mechanism by which the HCV p7 protein promotes viral assembly, focusing on its role in regulating the subcellular localization of the Core protein between LDs and the ER. Our data demonstrate that p7 delays Core maturation by interacting with SPP, thereby interfering with SPP-mediated cleavage of Core. We further identified a conserved leucine-rich motif within the C-terminal domain of p7 that is essential for SPP binding and inhibition of its proteolytic activity. Retention of the uncleaved, full-length Core at the ER enhances its interaction with the envelope glycoprotein E1, thereby promoting efficient viral particle assembly and release. Collectively, these findings establish a novel function of p7 in modulating Core processing and intracellular trafficking through direct interaction with SPP, contributing to optimal HCV morphogenesis.

## RESULTS

### p7 delays Core protein processing via interaction with signal peptide peptidase (SPP)

Previous studies have shown that HCV p7 protein promoted the localization of HCV Core protein to the endoplasmic reticulum (ER), with variations observed between different HCV subtypes such as JFH1 and JC1. Additionally, the presence of a p7-NS2 fusion protein affects Core localization in a subtype-dependent manner (14). However, the specific contribution of p7 to Core localization at the ER and its subsequent processing remains incompletely understood. To investigate this, we employed a transient expression system to assess whether p7 or NS2 modulates Core maturation. We constructed a full-length Core (fl-Core) derived from genotype 1a (H77S), tagged with an HA epitope at its C-terminus, allowing us to distinguish the fl-Core from mature Core (1–177 aa) (m-Core). Co-expression in HEK293T cells of fl-Core, NS2, and p7 revealed that p7 expression significantly inhibited the fl-Core processing to m-Core compared to vector control (Fig. 1A). This result suggests that p7 acts as a negative regulator of Core maturation. To assess whether this effect is conserved across HCV genotypes, we next analyzed p7 derived from the genotype 2a strain JFH1 and J6. Interestingly, JFH1 p7 also inhibited Core processing, although less efficiently than H77S p7 (Fig. 1B). Notably, J6 p7 exhibited a slightly higher capacity to induce fl-Core accumulation compared to JFH1 p7. Given that SPP mediates cleavage of fl-Core to m-Core, we hypothesized that p7 modulates Core maturation in a genotype-specific manner by differentially interacting with SPP.

Co-immunoprecipitation (co-IP) assays confirmed that both H77S and JFH1 p7 physically associate with endogenous SPP (Fig. 1C). Interestingly, the interaction between JFH1 p7 and SPP was weaker than that of H77S p7, potentially explaining the less efficient inhibitory effect of JFH1 p7 on Core maturation. To examine whether the ion channel activity of p7 is involved in this interaction, we used an ion channel–deficient mutant, p7 K33A-R35A [hereafter referred to as p7(KRAA)] (15). The ion channel-deficient mutant p7(KRAA) retained the ability to interact with SPP (Fig. 1D), indicating that ion channel deficiency does not impair the p7-SPP interaction. As expected, the effect of p7(KRAA) on Core protein processing was comparable to that of wild-type p7 (p7 WT) (Fig. 1E).

These findings demonstrate that p7 regulates Core protein processing through its interaction with SPP and that this association is maintained even in ion-channel deficient mutants.

### The L51-55 region of p7 is critical for interaction with SPP and regulation of Core processing

**Fig 2:**
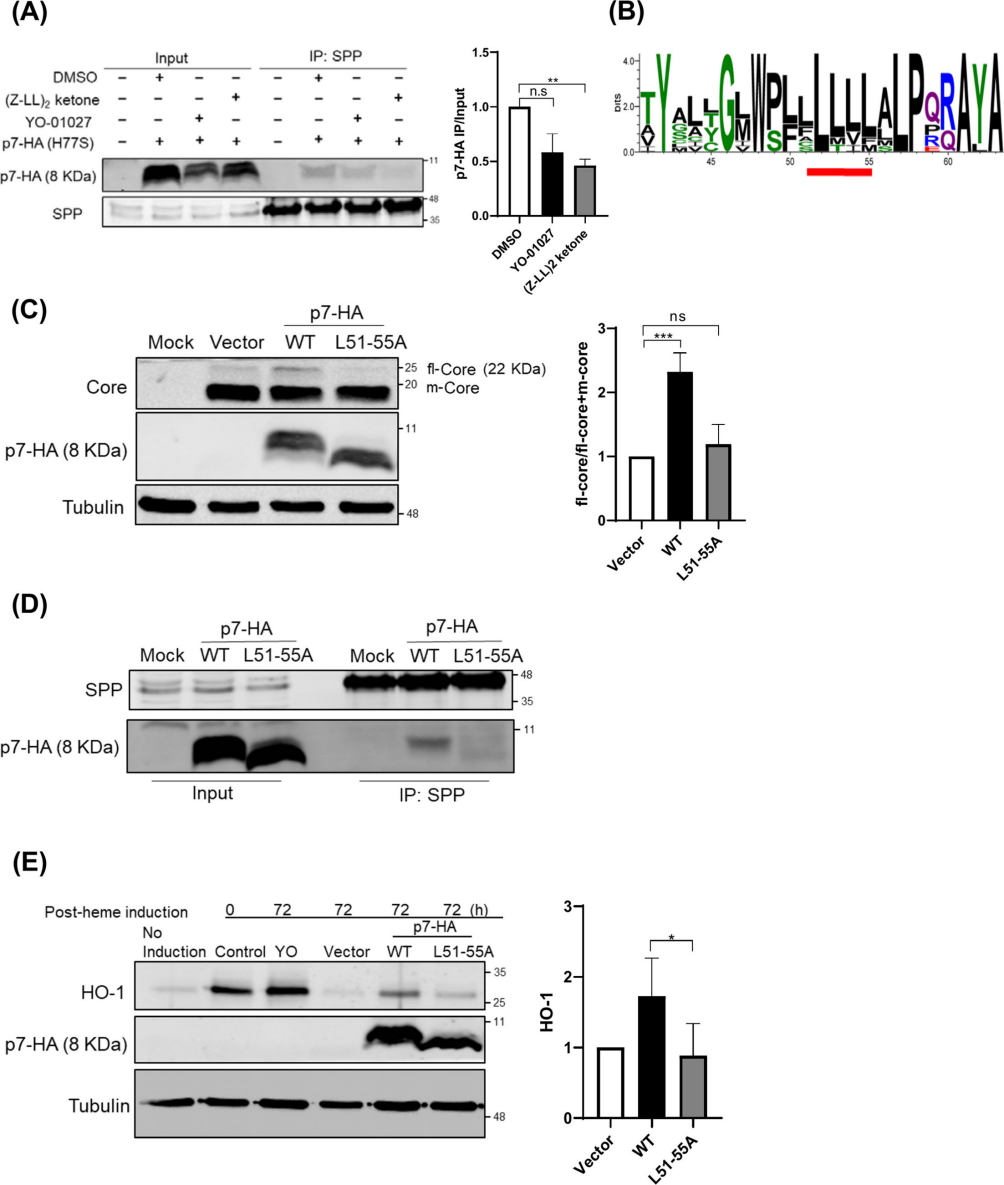
The L51-55 region of HCV p7 is critical for SPP interaction and regulation of Core processing. (**A**) FT3-7 cells expressing HA-tagged p7 (H77S) were treated with SPP inhibitors YO-01027 (Cat# 50-187-2905, Thermo Fisher) or (Z-LL)₂-ketone (Cat# 42-105-05MG, Millipore) at 5 h post-transfection. At 48 h, co-IP was performed to detect p7-SPP interaction by Western blot. (**B**) Sequence logo of HCV p7 showing amino acid conservation across genotypes. The conserved L51-55 region is highlighted in red. (**C**) Western blot analysis of Core processing with WT or L51-55A mutant p7-HA. fl-Core levels were quantified (right panel, mean ± SD, *n* = 3). (**D**) Co-IP of endogenous SPP with WT or L51-55A mutant p7-HA in FT3-7 cells. (**E**) FT3-7 cells transfected with WT or L51-55A p7-HA were treated with 50 µM hemin (16 h) to induce HO-1, with YO-01027 as a positive control for SPP inhibition. Cells were harvested 72 h post-treatment for immunoblotting (left) and HO-1 quantification (right). Data were normalized to vector control (*n* = 3, mean ± SD). Statistical significance was determined by unpaired *t*-test: *, *P* < 0.05; **, *P* < 0.01; ***, *P* < 0.001; ns = not significant (*P* > 0.05).

To further elucidate the mechanism by which p7 regulates Core processing, we tested whether SPP inhibition affects the interaction between p7 and SPP. FT3-7 cells expressing p7 were treated with two SPP inhibitors: γ-secretase inhibitor YO-01027 and (Z-LL)₂-ketone. YO-01027 inhibits SPP indirectly by targeting the N-terminal fragment of presenilin, a component of the γ-secretase complex (16), whereas (Z-LL)₂-ketone acts as a competitive inhibitor by mimicking the hydrophobic, leucine-rich regions of natural SPP substrates and binding to the catalytic site (17). (Z-LL)₂-ketone markedly reduced the interaction between p7 and SPP (Fig. 2A). In a sequence alignment of p7 across diverse HCV genotypes, we identified a highly conserved leucine-rich motif at the C-terminus encompassing residues L51-55 (Fig. 2B). The L51-55 motif, characterized by a highly conserved cluster of leucine residues, likely forms a critical hydrophobic interface within the ER membrane. Leucine-rich motifs are well-documented to facilitate protein-protein interactions by stabilizing trans-membrane alpha-helices and promoting the formation of specific hydrophobic contacts (18).

We hypothesized that the conserved L51-55 motif mediates SPP binding and regulates Core processing. To test this, we generated a p7 mutant in which leucines at positions 51–55 were replaced with alanines (L51-55A). As shown in Fig. 2C, the L51-55A mutant failed to inhibit Core processing compared to p7 WT. Co-immunoprecipitation assays further confirmed that, unlike p7 WT, the mutant did not bind to SPP (Fig. 2D). To assess whether p7 also affects host SPP substrates, we examined the stability of heme oxygenase 1 (HO-1), a known SPP-regulated protein (19). HEK293T cells transfected with either p7 WT or the L51-55A mutant were treated with hemin to induce HO-1 expression. While p7 WT delayed HO-1 degradation relative to vector control, the L51-55A mutant had no effect (Fig. 2E). Notably, although relative expression levels varied modestly across independent experiments, the inability of the L51-55A mutant to delay HO-1 degradation persisted even when expressed at higher levels than p7 WT, indicating that the defect is not attributable to protein abundance. Together, these results demonstrate that p7 modulates both viral and host SPP substrates and that the L51-55 motif is critical for its interaction with SPP and regulation of Core processing.

### The L51-55 motif of p7 is essential for infectious virus production

**Fig 3:**
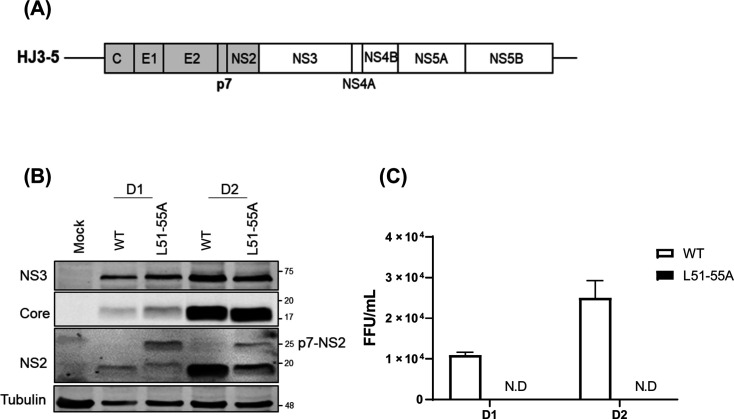
The p7 L51-55A mutation impairs p7-NS2 processing and HJ3-5 virus production. (**A**) Schematic of organization of infectious HCV (HJ3-5) encoding the Core-to-NS2 sequence from genotype 1a H77S (shaded) and the rest of the coding sequence from genotype 2a JFH1 (open). (**B**) Western blot of Core, NS2, NS3, and Tubulin in FT3-7 cells at indicated time points post-electroporation with WT or L51-55A HJ3-5 RNA. (**C**) Extracellular viral titers measured by focus-forming assay at indicated times.

**Fig 4:**
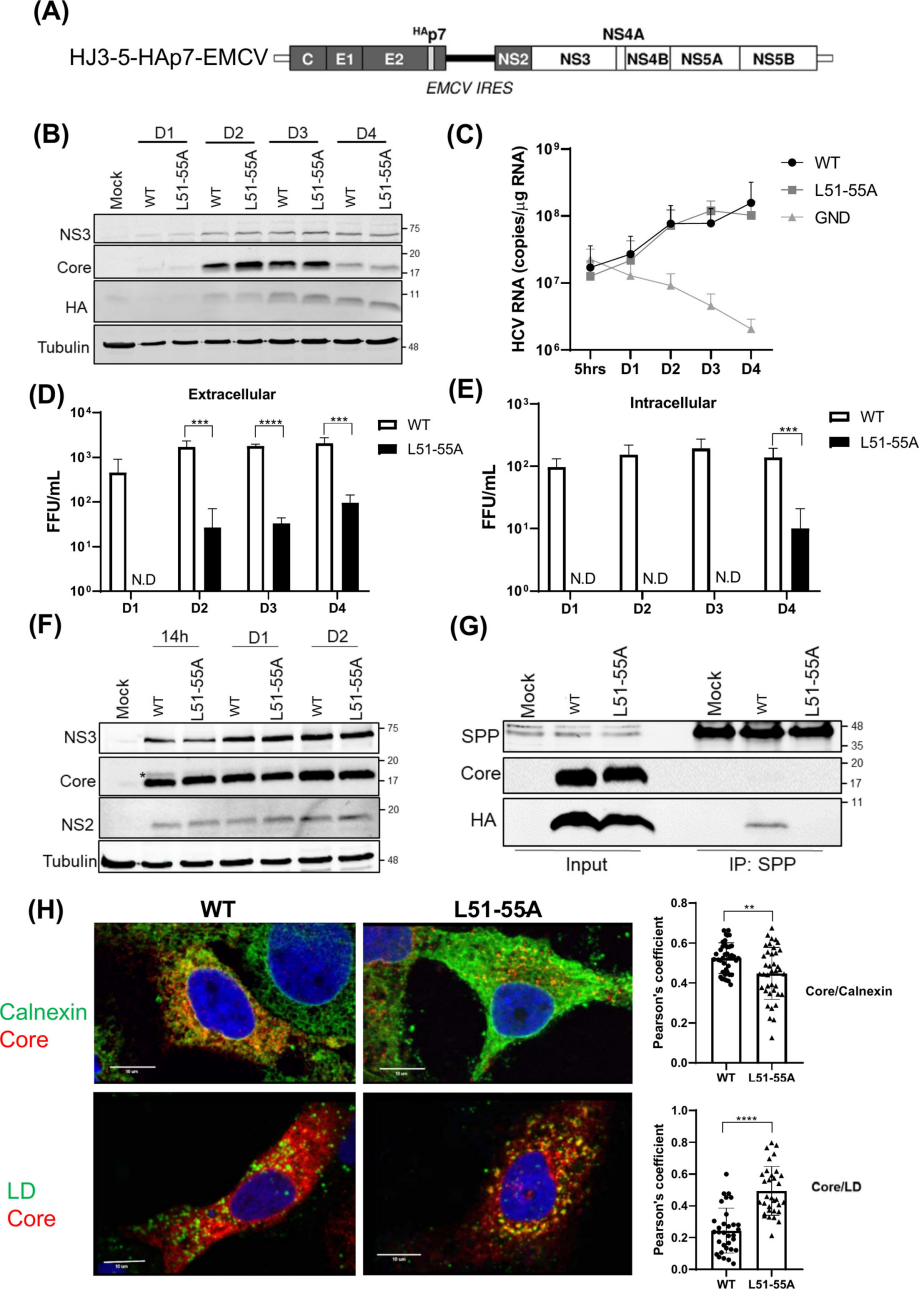
The p7 L51-55A mutation impairs virus assembly by disrupting SPP interaction and accelerating Core processing. (**A**) Schematic of modified HJ3-5 RNA encoding N-terminal HA-tagged p7 and EMCV IRES between p7 and NS2. (**B**) Western blot of Core, HA, NS3, and Tubulin in FT3-7 cells at indicated time points post-electroporation with WT or L51-55A HAp7-EMCV RNA. (**C**) Intracellular HCV RNA quantified by RT-qPCR (fold change relative to 5 h). (**D and E**) Extracellular (**D**) and intracellular (**E**) viral titers measured by focus-forming assay at indicated times. (**F**) Western blot of Core, NS2, NS3, and Tubulin in Huh7.5 cells at early time points. * means fl-Core (**G**) Co-IP assay of HA-tagged p7-SPP interaction in Huh7.5 cells expressing WT or L51-55A HAp7-EMCV RNA. (**H**) Confocal microscopy (left) and Pearson’s correlation analysis (right) showing colocalization of Core with lipid droplets (LD, top) or calnexin (ER marker, bottom) in Huh7.5 cells at 14 h post-electroporation. These measurements were derived from 30 cells across 3 independent experiments. Graphs show mean ± SD (*n* = 3). Statistical significance: ****, *P* < 0.0001; ***, *P* < 0.001; **, *P* < 0.01, ND, not detected.

To assess the functional role of the p7-SPP interaction during HCV assembly, we introduced the p7 L51-55A mutant into the HJ3-5 plasmid (Fig. 3A). As shown in Fig. 3B, this mutation disrupted proper processing at the p7-NS2 junction, leading to the accumulation of a p7-NS2 fusion protein and consequent reduction in viral production (Fig. 3C). To overcome this, we engineered a modified HJ3-5 genome (HJ3-5/HAp7-EMCV) in which an HA tag (hemagglutinin) was inserted at the N-terminus of p7 and an encephalomyocarditis virus (EMCV) internal ribosome entry site (IRES) was introduced between p7 and NS2 (Fig. 4A). This design uncouples p7 from NS2 while preserving its expression, thereby avoiding unintended effects on RNA replication. We then introduced the HJ3-5/HAp7 L51-55A mutation into this construct to assess its effect on viral replication and assembly.

Following electroporation into cells, we observed that the L51-55A mutation did not significantly affect intracellular HCV RNA accumulation or expression of viral proteins, as demonstrated by Western blot and quantitative real-time PCR (qRT-PCR) analysis (Fig. 4B and C). However, both extracellular and intracellular infectious virus titers were markedly reduced in cells electroporated with the L51-55A mutant RNA compared to the WT control (Fig. 4D and E). While the L51-55A mutation markedly reduced viral production, a low level of extracellular infectivity (~20–30 FFU/mL) remained detectable at day two post-electroporation (Fig. 4D). In contrast, intracellular infectious particles for this mutant were below our assay’s limit of detection at the same time point. Intracellular viral titers are typically approximately 10-fold lower than those found in the supernatant. Consequently, the low levels of assembly supported by the L51-55A mutant only reached the threshold of intracellular detection by day 4, as viral particles gradually accumulated within the cell. These results suggest that while p7-SPP interaction is critical for efficient assembly, the L51-55A mutant retains a baseline level of viral production that is initially only capturable in the extracellular fraction due to its higher relative titer. We observed that fl-Core was barely detectable in virus-replicating WT cells during the time course, likely due to rapid proteasomal degradation (20). To address this, we performed a time-course analysis in Huh7.5 cells, which support higher levels of early-stage viral replication, rather than using proteasomal inhibitors that might interfere with viral replication. As shown in Fig. 4F, fl-Core was detectable at 14 h post-electroporation in WT cells but absent in cells electroporated with the L51-55A mutant. Co-immunoprecipitation analysis further confirmed that p7 interacts with endogenous SPP in virus-replicating cells, while the L51-55A mutant fails to do so (Fig. 4G), consistent with the protein expression system (Fig. 2D). To investigate whether impaired Core processing affected subcellular localization, we performed confocal microscopy followed by quantitative colocalization analysis. In Huh7.5 cells electroporated with the L51-55A mutant, Core showed increased colocalization with lipid droplets (LDs) (average Pearson’s correlation coefficient: 0.494 for mutant vs 0.244 for WT; Fig. 4H, upper panel), while colocalization with the ER marker calnexin was reduced (Pearson’s *r* = 0.448 for mutant vs 0.525 for WT; Fig. 4H, lower panel). Collectively, these results demonstrate that the conserved L51-55 motif in p7 mediates its interaction with SPP, which is essential for proper Core processing, ER localization, and efficient HCV particle production.

### Full-length Core interacts with E1 to promote efficient HCV particle production

**Fig 5:**
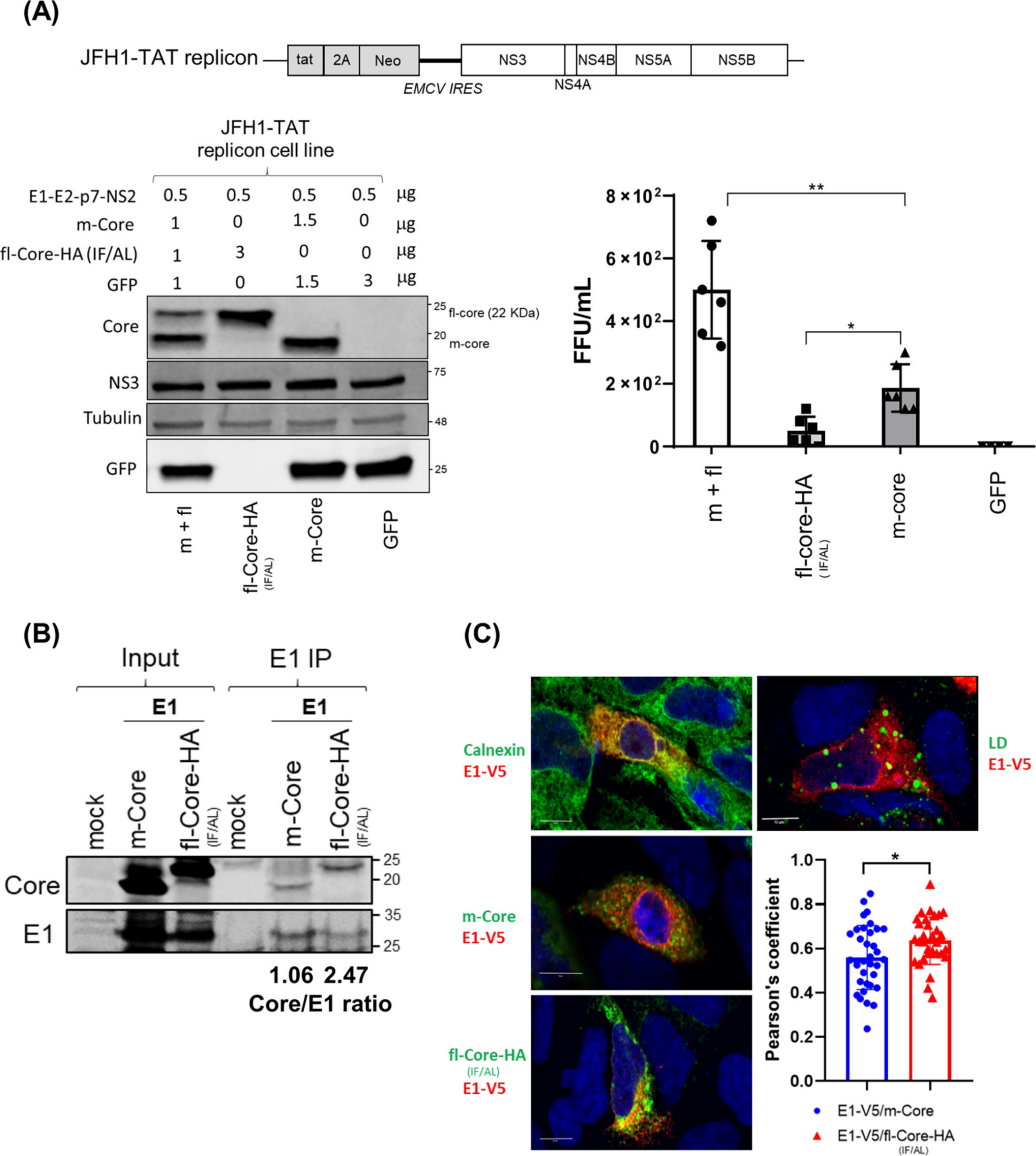
Full-length Core enhances HCV particle production through interaction with E1. (**A**) Schematic of JFH1-TAT subgenomic replicon. JFH1-TAT replicon cells were transfected with E1-E2-p7-NS2 and various ratios of m-Core, full-length Core-HA (IF/AL), or GFP. At 24 h, Western blot shows protein expression (left); corresponding viral titers are shown (right). (**B**) 293T cells transfected with E1E2, m-Core, or fl-Core-HA (IF/AL). Co-IP was performed with anti-E1 in the presence of transfer RNA (0.1 mg/mL) to assess Core-E1 interaction. (**C**) Representative confocal microscopy images of Huh7.5 cells at 24 h post-transfection. Top panels show the colocalization of E1-V5 (red) with the ER marker calnexin (green, left) or lipid droplets (LD, green, right). Bottom panels illustrate the colocalization of E1-V5 (red) with either m-Core (green) or fl-Core-HA (IF/AL) (green). (Right) Pearson’s correlation coefficient analysis quantifying the colocalization between E1-V5 and the respective Core constructs. Data represent measurements from 30 cells across 3 independent experiments. Statistical significance: **, *P* < 0.01; *, *P* < 0.05.

Our findings suggest that p7 delays Core processing, resulting in the accumulation of fl-Core. To assess the functional role of fl-Core for HCV particle production, we employed the JFH1-TAT subgenomic replicon system, which supports RNA replication but lacks structural genes and is incapable of producing infectious particles. To determine if virus production could be rescued, we investigated whether exogenous expression of full-length Core (fl-Core) or mature Core (m-Core) could circumvent the p7-dependent maturation step and restore the assembly of infectious particles. FT3-7 cells were co-transfected with plasmids encoding structural proteins, including Core constructs and E1-E2-p7-NS2 (Fig. 5A). We tested two forms of Core: fl-Core tagged with a C-terminal HA tag—including an IF/AL mutant resistant to SPP cleavage (11) and m-Core, mimicking the SPP-processed product. Core variants were transfected at different ratios, and protein expression was confirmed by Western blot (Fig. 5A, left panel). Supernatants were collected 24 h post-transfection, and viral titers were determined by focus-forming assay.

As shown in Fig. 5A, right panel, co-expression of both fl- and m-Core significantly enhanced viral production compared to either form alone, suggesting a synergistic role in virus assembly. Given prior evidence of Core-E1 interactions (21), we hypothesized that fl-Core potentially associates more strongly with E1 compared to m-Core. To test this, we performed co-immunoprecipitation using an anti-E1 antibody in HEK293T cells co-expressing E1-E2 and either fl-Core or m-Core. As shown in Fig. 5B, fl-Core exhibited a significantly stronger interaction with E1 compared to m-Core. To further characterize the spatial relationship between Core and E1 in an exogenous expression system, we performed confocal microscopy in Huh7.5 cells. We first examined the subcellular distribution of E1-V5, which demonstrated significant colocalization with the ER marker calnexin, but not with LDs. We then compared the colocalization patterns of E1-V5 with two different Core constructs: m-Core and the fl-Core-HA (IF/AL). While both forms of Core showed proximity to E1, the fl-Core-HA (IF/AL) exhibited a higher degree of colocalization with E1-V5 compared to m-Core. Quantitative analysis using Pearson’s correlation coefficient confirmed a statistically significant increase in colocalization for the E1-V5/fl-Core-HA (IF/AL) pair relative to the E1-V5/m-Core pair. Although E1-Core association was not directly visualized at lipid droplets, ER-based co-localization analyses indicate that fl-Core preferentially interacts with ER-resident E1 prior to Core maturation and lipid droplet targeting. These findings suggest that the delayed processing of Core, mediated by p7, leads to the accumulation of fl-Core, which facilitates both the interaction and colocalization of Core with E1.

This highlights a previously unrecognized role for fl-Core in HCV morphogenesis and reveals that p7 supports viral assembly not only by delaying Core maturation but also by promoting productive Core-E1 interactions.

## DISCUSSION

**Fig 6:**
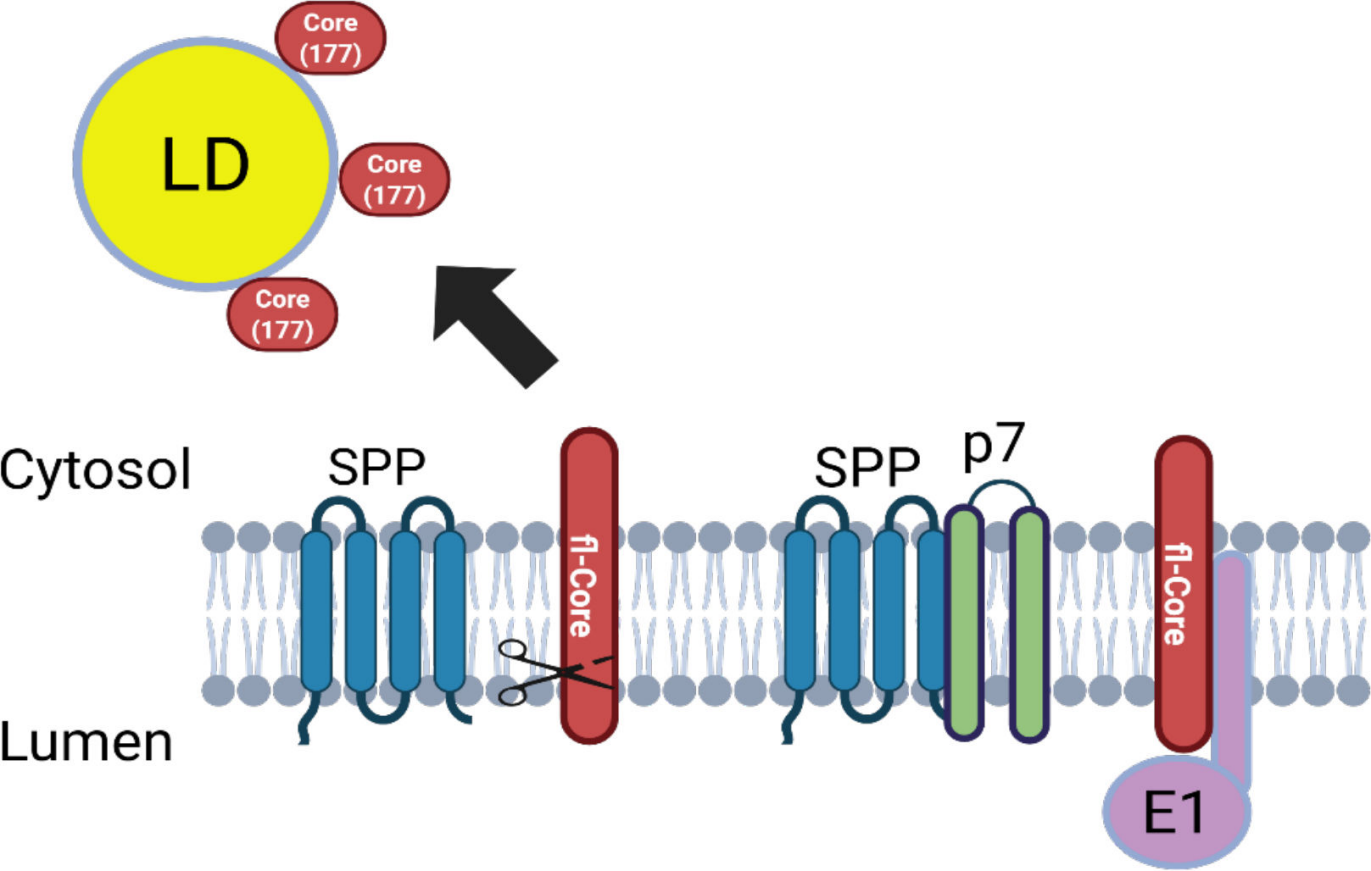
Model of p7 function in HCV assembly. p7 interacts with SPP to modulate Core processing. In the presence of p7, SPP-mediated cleavage of full-length Core is reduced, leading to its accumulation at the endoplasmic reticulum (ER) membrane. Retention of unprocessed full-length Core at the ER promotes interaction with the E1 glycoprotein, facilitating efficient virus particle assembly.

This study highlights the critical role of p7 in regulating the processing of HCV Core protein by host signal peptide peptidase (SPP), thereby influencing Core subcellular localization and enhancing its interaction with envelope protein E1—both of which are essential for efficient viral assembly and infectious virus production (Fig. 6).

We identified a conserved leucine-rich motif (L51-55) in the C-terminal domain of p7 as a key determinant for SPP binding. This region is essential for the p7-SPP interaction, which modulates Core protein maturation. Previous studies have shown mutations within this motif reduce p7 sensitivity to amantadine and modestly impair oligomerization (21). Our findings suggest a mechanistic link between p7 oligomer formation and its ability to engage host factors such as SPP. It is plausible that p7 adopts an oligomeric state to create a functional binding interface for SPP. Interestingly, comparison of the leucine-rich region across HCV genotypes revealed that genotype 1a (H77S) contains the highest leucine content at the residues 51-55 motif (LLLLL), while genotype 2a (J6) carries SLLLL, and genotype 2a (JFH1) carries CLLLM.

Correspondingly, we observed that J6 p7 induced a higher accumulation of fl-Core compared to JFH1 p7 (Fig. 1B). This suggests that the higher leucine density in the J6 L-rich motif may enhance its regulatory effect on Core maturation, potentially through a stronger association with SPP, similar to what was observed with the H77S strain. Indeed, our co-immunoprecipitation data confirmed that JFH1 p7, which has the lowest leucine content among the three, exhibits a weakened interaction with SPP compared to H77S (Fig. 1C). Moreover, mutation of this region not only abolished the p7-SPP interaction but also disrupted processing at the p7-NS2 junction, resulting in the accumulation of p7-NS2 fusion proteins (Fig. 3B). This defect in p7-NS2 processing abrogated viral particle production (Fig. 3C), underscoring the multifunctional role of the L51-55 motif in both p7 maturation and its interaction with host factors critical for the viral life cycle.

Carrère-Kremer et al. (22) reported that the inefficient cleavage of junction sites around p7 is likely due to structural constraints at the E2-p7 and p7-NS2 boundaries. Notably, delayed or suboptimal processing at the E2-p7 junction appears to be essential for efficient virus assembly. Disruption of this finely tuned cleavage—whether by introduction of mutations, epitope tags, or insertion of an encephalomyocarditis virus (EMCV) internal ribosome entry site (IRES)—leads to impaired HCV assembly (23). These findings suggest that timely release of p7 from its polyprotein precursor is critical for efficient HCV assembly. Based on our data, we propose that p7 engages SPP at a later stage of infection to retain full-length Core in the endoplasmic reticulum, thereby promoting Core-E1/E2 interactions and facilitating virion assembly.

We observed that the SPP inhibitors (Z-LL)₂-ketone and YO-01024, which target the protease’s catalytic center, significantly disrupted the p7-SPP association (Fig. 2A). This displacement suggests that the p7-SPP interface either overlaps with, or is conformationally coupled to, the SPP active site, supporting a model of direct functional coupling between p7 and the SPP catalytic machinery. While these data argue against a purely spatial mechanism, they do not exclude the possibility that p7 concurrently alters the subcellular context in which Core processing occurs. Indeed, a synergistic model may exist in which p7-mediated modulation of ER membrane properties facilitates the recruitment or retention of Core and SPP within specific microdomains, thereby enabling efficient processing through direct engagement with SPP.

A key observation in our study is the preferential interaction of E1 with fl-Core over its mature form (m-Core), despite both forms sharing the established E1-binding domain (aa 72–91) (21). Our data suggest that this preference is not governed by primary sequence differences but rather by spatial coordination and subcellular compartmentalization during the viral assembly process. We propose that the C-terminal signal peptide of fl-Core serves as a critical topological determinant. By anchoring fl-Core to the ER membrane, this hydrophobic tail likely constrains the aa 72–91 region in a specific orientation that is stereochemically favorable for interaction with the E1 cytoplasmic domain. In contrast, the processing of fl-Core into m-Core triggers a rapid translocation of the protein to lipid droplets (LDs). This translocation effectively segregates m-Core from the ER-resident E1 pool, as supported by our Pearson’s correlation analysis showing significantly reduced colocalization between m-Core and E1 (Fig. 5C).

Furthermore, our findings highlight a crucial temporal window for assembly. The p7-mediated delay in Core processing ensures that a sufficient pool of fl-Core remains at the ER membrane to engage E1. This kinetic regulation likely optimizes the efficiency of viral envelope acquisition before the Core protein is sequestered to LDs. Thus, the p7-Core-E1 axis represents a highly coordinated mechanism where protein processing kinetics and spatial localization converge to facilitate infectious particle production.

In our study, we observed that p7 from genotype 1a (H77S) exhibited a more robust effect on Core processing compared to p7 from genotype 2a (JFH1). While our co-IP data in Fig. 1C suggests that this difference is associated with a reduced interaction between JFH1 p7 and the SPP, the potential impact of using a heterologous system should be considered. Since we utilized the Core protein derived from the H77S strain throughout the study, the relatively lower efficiency of JFH1 p7 might also stem from suboptimal compatibility between the genotype 1a Core and genotype 2a p7 sequences. It is possible that the protein-protein interaction interfaces involved in the recruitment of processing enzymes are more finely tuned within a homologous genotype context. Therefore, while our results highlight p7-SPP interaction as a key factor, we cannot entirely rule out the influence of genotype-specific interactions between Core and p7 on polyprotein processing. Further studies using fully homologous systems for various genotypes will be beneficial to clarify these relative contributions.

In summary, our findings identify the L51-55 region of p7 as essential for SPP binding and proper cleavage of the p7-NS2 junction—both of which are critical for infectious HCV production. Furthermore, we show that full-length Core exhibits enhanced affinity for the E1 glycoprotein, supporting a coordinated mechanism by which p7 controls Core maturation and promotes efficient virion assembly.

## MATERIALS AND METHODS

### Cell culture

HEK293T and clonal derivative of Huh7, including Huh7.5 (kindly provided by Dr. Charles Rice at the Rockefeller University [24]), and FT3-7 (25) cells were cultured in Dulbecco’s Modified Eagle’s Medium (DMEM) containing 10% fetal bovine serum (Invitrogen, Carlsbad, CA, USA) at 37°C under a humidified atmosphere with 5% CO_2_. JFH1-TAT replicon cell was cultured in 10% FBS in DMEM containing 0.5 mg/mL G418 for maintaining HCV replication (26).

### Plasmid construction

Plasmids encoding HCV Core with an N-terminal Flag and C-terminal HA tag were amplified from HCV genotype 1a (H77S) (27). A plasmid expressing the truncated HCV Core (residues 1–177) was generated by amplifying the corresponding fragment and cloning it into an expression vector. To generate fl-Core-HA (IF/AL), we utilized a Core construct containing a C-terminal HA tag and the IF176-177AL (IF/AL) mutation, which is designed to prevent cleavage by SPP (11). Site-directed mutagenesis was performed to introduce these amino acid substitutions using the following primers: Forward: 5′-CCTGGTTGCTCTTTCTCTGCCTTACTTCTGGCCCTGCTCTCT-3′; Reverse: 5′-AGAGAGCAGGGCCAGAAGTAAGGCAGAGAAAGAGCAACCAGG-3′. The complementary DNA (cDNA) encoding HCV NS2 with an N-terminal Flag tag (DYKDDDDK) was amplified from the H77S strain and cloned into the pcDNA6/V5-His vector (Invitrogen, Carlsbad, CA, USA), as previously described (28). For p7 expression, cDNAs encoding HCV p7 with a C-terminal HA tag were amplified from both H77S and genotype 2a (JFH1 and J6), and cloned into pcDNA6/V5-His to generate p7-HA constructs. The p7(KRAA) mutations were introduced into the indicated plasmids by PCR-based mutagenesis. To create the L51-55A mutant in (p7-HA/L51-55A), site-directed mutagenesis was carried out using the following primer set: forward, 5′-CTACGGGATGTGGCCTCTCGCCGCGGCCGCGGCGGCGTTGCCTCAGCG-3′; reverse, 5’ CGCTGAGGCAACGCCGCCGCGGCCGCGGCGAGAGGCCACATCCCGTAG-3′.

For E1-E2-p7-NS2 plasmid expression was described previously (26). The infectious HCV clone HJ3-5/HAp7-EMCV-NS2 was constructed by inserting the encephalomyocarditis virus (EMCV) internal ribosome entry site (IRES) between the HA-tagged p7 and NS2 regions of the HJ3-5 background (29). The L51-55A mutation was introduced into this construct to generate HAp7/L51-55A-EMCV-NS2 by using primers described above. All mutations were introduced by using the QuikChange II XL Site-Directed Mutagenesis Kit (Agilent Technology, Santa Clara, CA, USA). To express the E1-V5 plasmid, the E1 sequence was amplified from HCV genotype 1a (H77S), and a V5 tag was fused to the sequence during PCR amplification. All plasmid constructs were verified by Sanger sequencing of the manipulated regions.

### Transfection of expression plasmids

All plasmid expression experiments were performed using JetOPTIMUS transfection reagent (Sartorius, New York, USA) according to the manufacturer’s instructions.

### The quantitative real-time RT-PCR assay

Intracellular HCV RNA was isolated by using an RNeasy RNA isolation kit (Qiagen, Valencia, CA, USA). To quantitate the level of HCV RNA, a real-time RT-PCR assay was performed by using a QuantiNova Probe RT-PCR Kit (Qiagen, Valencia, CA, USA) and a CFX96 Real-Time System (Bio-Rad, Hercules, CA, USA) with custom-designed primer-probe sets (forward primer HCV84FP, 5′-GCCATGGCGTTAGTATGAGTGT-3′; reverse primer HCV 303RP, 5′-CGCCCTATCAGGCAGTACCACAA-3′; and probe HCV146BHQ, FAM-TCTGCGGAACCGGTGAGTACACC-DBH1) as described in detail before (26).

### Western blot analysis

Cell lysates were prepared in a lysis buffer (50 mM Tris-HCl, 150 mM NaCl, 1% Triton X-100, 0.5% sodium deoxycholate, pH 7.5, and 2 mM EDTA) containing 1× protease and phosphatase inhibitor cocktail mix (GenDEPOT, Katy, TX, USA), separated by SDS-PAGE and transferred onto polyvinylidene difluoride membranes. The membrane was blocked and probed with primary antibodies to Core (1:2,000 dilution of C7-50, Thermo Scientific, Rockford, IL, USA), E1 (1:1,000 dilution, Cat# MBS310203, MyBioSource), NS2 (polyclonal rabbit anti-NS2 antibody) (30), NS3 (1:2,000 dilution of 9-G2, ViroGen, Watertown, MA, USA), HA (1:2,000 dilution of C29F4, Cell Signaling Danvers, MA, USA), V5 (Cat# D3H8Q and E9H8O, Cell Signaling, Danvers, MA, USA), Tubulin (1:7,000 dilution, EMD Millipore, Billerica, MA, USA), signal peptide peptidase (SPP) (1:2,000 dilution, Cat# ab190253, Abcam), and HO-1 (1:2,000 dilution of 10701-1-AP, Proteintech). Protein bands were visualized by incubating the membranes with IRDye Secondary Antibodies (Li-Cor Biosciences, Lincoln, NE, USA), followed by imaging with an Odyssey Infrared Imaging System (Li-Cor Biosciences, Lincoln, NE, USA).

### *In vitro* HCV RNA transcription and electroporation

HCV cDNA was linearized with Xba-I (NEB, Hitchin, United Kingdom), followed by transcription to RNA by using a T7 Megascript Kit (Ambion, Austin, TX, USA). HCV RNA was purified by using an RNeasy RNA Isolation Kit (Qiagen, Valencia, CA, USA). For electroporation, 10 µg of RNA was mixed with 5 × 10^6^ FT3-7 cells in a 0.4 cm gap width electroporation cuvette (Bio-Rad, Hercules, CA, USA) and pulsed once at 270V and 950 µF by using a GenePulser system (Bio-Rad, Hercules, CA, USA).

### HCV infectivity assays

For virus titration, 50 µL aliquots of serial 10-fold dilutions of cell culture supernatant fluids, clarified by low-speed centrifugation, were inoculated onto naïve Huh-7.5 cells in a 96-well plate. After 72 h, cells were fixed with 4% PFA for 20 min and then stained with anti-Core antibody (Affinity BioReagents; 1:1,000). For intracellular virus titration, cells were trypsinized, resuspended in a 500 µL medium, and lysed by four cycles of freeze-thawing. Clarified cell lysates were used to inoculate Huh-7.5 cells as described above. HCV infectivity was determined by counting the clusters of Core-immunostained cells (focus), and the titer was expressed as focus-forming units per milliliter (FFU/mL).

### Co-immunoprecipitation (co-IP) assays

Cells were transfected with various plasmids and harvested for further assays 48 h after transfection. Transfected cells were washed twice with phosphate-buffered saline (PBS) and scraped into lysis buffer (Cat#9803, Cell Signaling Technology, Danvers, MA, USA) with freshly added containing 1× protease inhibitor (GenDepot, Katy, TX, USA). After incubation for 30 min on ice, the lysate was centrifuged to remove the insoluble cell debris. Equal amounts of lysates were used for the co-IP assay by incubating overnight with 0.5 µg of an anti-SPP antibody or anti-E1 antibody at 4°C. After incubation, add Dynabeads Protein G (Invitrogen) 10 µL for another incubation 2 h at room temperature. Wash Dynabeads with cold-PBS twice. The immune complexes were denatured by 37°C for 20 min. Samples were then resolved by 14% SDS-PAGE and analyzed by Western blotting.

### Confocal microscopy

Electroporated cells were plated on eight-well chamber slides (BD Biosciences, Bedford, MA, USA) at a density of 1 × 10^4^ cells per well. Two days later, electroporated cells were fixed in 4% paraformaldehyde. After blocking non-specific binding by incubating the cells in a PBS solution containing 3.5% bovine serum albumin for 1 h at room temperature, cells were incubated with primary antibodies for 2 h and then with secondary antibodies for 1 h at room temperature. The slides were examined with an Olympus FluoView FV10i confocal microscope (Olympus America Inc., Waltham, MA, USA). Pearson’s coefficient was obtained by using FV10i-ASW 4.2 viewer software.

### Statistical analyses

Student’s *t*-test (unpaired) was performed by using GraphPad Prism version eight softwarwe to determine the significance in differences between paired values from at least three independent experiments. A *P*-value less than 0.05 was considered statistically significant.

## Data Availability

The data supporting this study’s findings are available from the corresponding author upon reasonable request.
